# Spiking Pattern of the Mouse Developing Inner Hair Cells Is Mostly Invariant Along the Tonotopic Axis

**DOI:** 10.3389/fncel.2018.00407

**Published:** 2018-11-14

**Authors:** Anne-Gabrielle Harrus, Jean-Charles Ceccato, Gaston Sendin, Jérôme Bourien, Jean-Luc Puel, Régis Nouvian

**Affiliations:** Institut des Neurosciences de Montpellier (INM), Inserm, University of Montpellier, Montpellier, France

**Keywords:** cochlea, sensory cells, action potential, calcium transients, spontaneous activity

## Abstract

During development, the sensory cells of the cochlea, the inner hair cells (IHCs), fire spontaneous calcium action potentials. This activity at the pre-hearing stage allows the IHCs to autonomously excite the auditory nerve fibers and hence, represents an efficient mechanism to shape the tonotopic organization along the ascending auditory pathway. Using calcium imaging, we show that the activity in the developing cochlea consists of calcium waves that propagate across the supporting and sensory cells. Both basal and apical IHCs were characterized by similar spontaneous calcium transients interspaced with silent periods, consistent with bursts of action potentials recorded in patch-clamp. In addition, adjacent auditory hair cells tend to have a synchronized [Ca^2+^]_i_ activity, irrespective of their location along the base-to-apex gradient of the cochlea. Finally, we show that the mechanical ablation of the inner phalangeal cells (IPCs), a class of supporting cells, reduces the synchronized [Ca^2+^]_i_ activity between neighboring sensory cells. These findings support the hypothesis that the tonotopic map refinement in higher auditory centers would depend on the synchronization of a discrete number of auditory sensory cells.

## Introduction

The auditory sensory cells of the cochlea, the inner hair cells (IHCs), undergo a developmental change from generating action potential to gradual receptor potential (Kros et al., 1998; Beutner and Moser, 2001; Marcotti et al., 2003; Marcotti, 2012). Developing IHCs fire bursts of calcium action potentials in a spontaneous manner (Tritsch et al., 2007; Tritsch and Bergles, 2010; Johnson et al., 2011, 2013; Sendin et al., 2014), while after the onset of hearing (i.e., beginning of the second postnatal week, Mikaelian et al., 1965; Ehret, 1985), the expression of a large-conductance potassium channel prevents the action potential firing and enables the IHCs to generate a receptor potential (Kros et al., 1998). At this stage, IHCs are fully able to translate incoming sound stimulation into glutamate release onto the afferent auditory fibers (Nouvian et al., 2006).

During developmental stages, the secretion of glutamate in response to acoustic cues is not possible because of the immature state of the ear, i.e., closed ear canal and lack of mature endolymph prevent the incoming sound stimulation to activate the sensory hair cells (Anniko et al., 1979; Saunders et al., 1993). The spontaneous spiking activity at the pre-hearing stage allows the IHCs to excite the auditory nerve fibers (Beutner and Moser, 2001; Tritsch et al., 2010; Wong et al., 2013). Thus, it represents a valuable mechanism to specify the sensory neurons identity without sound-stimulation (Shrestha et al., 2018; Sun et al., 2018). In addition, the temporal pattern of the developing hair cells activity is crucial for the tonotopic map formation in the nuclei of the higher auditory centers (Clause et al., 2014, 2017). Up to now, two different mechanisms have been proposed to instruct the tonotopic map refinement. In a first scenario, the firing pattern in hair cells varies along the cochlea, i.e., basal and apical IHCs fire in a sustained and burst manner, respectively (Johnson et al., 2011). In this hypothesis, the tonotopic map maturation in the auditory centers would depend on continuous or interrupted inputs that arise from the cochlea. An alternative scenario consists in a homogenous firing pattern along the tonotopic axis (Sendin et al., 2014). In this case, the organization of the higher auditory centers would depend on synchronized activity of limited numbers of neighboring IHCs (Tritsch et al., 2007; Tritsch and Bergles, 2010; Wang et al., 2015). Here, we used calcium imaging in the immature cochlea to gain insight on the pattern of the IHCs spiking activity. Our results show that the IHCs activity pattern is mostly invariant along the tonotopic axis of the cochlea.

## Materials and Methods

### Animals

Swiss mice (*n* = 36) of either sex (Janvier Labs) were bred and handled in accordance with the animal welfare guidelines 2010/63/EC of the European Communities Council Directive. This study was carried out in accordance with the recommendations of the ministère de l’agriculture, de l’alimentation, de la pêche, de la ruralité et de l’aménagement du territoire (Housing Agreement B34-172-36, Experimental Agreement A34-507). The protocol was approved by the Comité d’éthique en expérimentation animale Languedoc-Roussillon N°36 (Project number APAFIS#6235).

### Calcium Imaging

After cervical dislocation [postnatal day 1 (P1) to 7 (P7)], apical or basal coils of the cochlea were dissected using the following extracellular solution (in mM): 5.36 KCl, 141.7 NaCl, 1 MgCl_2_-6H_2_O, 0.5 MgSO_4_–7H_2_O, 10 HEPES and 10 D-glucose (pH was adjusted to 7.2 with NaOH and the solution had an osmolarity between 290 mosmol/l and 310 mosmol/l). The preparation was then continuously superfused with an extracellular solution containing (in mM): 144 NaCl, 1.3 CaCl_2_, 5.8 KCl, 0.9 MgCl_2_, 10 HEPES, and 10 D-glucose (pH was adjusted to 7.2 with NaOH and the extracellular solution had an osmolarity between 290 mosmol/l and 310 mosmol/l). All chemicals were obtained from Sigma. To enable a better loading of the calcium dye inside the IHCs, tectorial membrane, outer hair cells (OHCs) and pillar cells were mechanically removed using glass micropipettes controlled by a micromanipulator (PatchStar, Scientifica) under an upright microscope (AxioExaminer, Zeiss). For experiments in which the inner phalangeal cells (IPCs) were removed, the glass micropipette was first sealed onto the IPC, a negative pressure was then applied and the pipette was removed, leading to the mechanical disruption of the IPC. Then, the preparation was incubated with Fura-2 AM (10 μm, Thermo Fisher Scientific) and pluronic acid (0.04%, Thermo Fisher Scientific) during 30 min and followed by a washout of 10 min. The microscope was equipped with a Filter set 21 HE (Zeiss) using the following filters/dichroic: Excitation BP: 340/30 and BP: 387/15, Beamsplitter FT: 409 and Emission BP: 510/90. Fura-2 was excited at 350/380 nm using a polychrome V (TILL-photonics). Emission fluorescence was imaged with a CDD camera (Orca-R^2^, Hamamatsu) using a ×63 water immersion objective (NA: 1, Zeiss). 2 × 2 (pixel size of 0.215 μm) or 4 × 4 (pixel size of 0.43 μm) binning was used together with long exposure times (100 ms) at a sampling frequency of 1 Hz, and emitted light was calculated from background-corrected fluorescence. Ratio (R) corresponds to the f350/f380 emitted light and ΔR = R(t) − R_0_, where t is the time and R_0_ corresponds to baseline.

### Analysis of the Calcium Activity

Regions of interest (ROIs) of approximately 20 μm^2^ were set at the center of IHCs, easily discernable by their shape and stereocilia. In order to remove a slow increase in the intracellular calcium baseline of the IHCs, which was sometimes observed during the time course of the experiment, and to remove the high-frequency noise components without altering the calcium transients waveforms, ratio was first filtered using a band-pass filter (Butterworth [0.005–0.1 Hz]). For each sample, the whole filtered calcium signals (residual noise plus transients) from the ROIs set in the IHCs were then averaged to obtain a grand standard deviation (SD) average that we used as a detection threshold. Therefore, calcium transients in IHCs were then detected when the ratio exceeds 1 SD. The duration of a calcium transient corresponds to the time interval between the onset and the return to baseline of the calcium transient, i.e., the time points when the calcium fluorescence intercepts one SD. In contrast to the hair cells, the inner supporting cells (ISCs) within the Kölliker’s organ were not individually identified. Rather, the ISCs area was divided into several ROIs of 81 μm^2^. This allow us to track the intracellular calcium transients in different locations of the ISC’s area. Here again, the calcium signals from the ROIs set in the Kölliker’s organ were filtered using a band-pass filter (Butterworth [0.005–0.1 Hz]) and then were averaged to obtain a grand SD average that we used as a detection threshold. Synchronization index (SI) between two ROIs was calculated as the ratio between the number of temporally overlapping calcium transients (Matlab logical “and” function) over the sum of overlapping plus non-overlapping calcium transients (Matlab logical “or” function). Thus, SI of 1 means that all the calcium transients in two ROIs temporally overlap, while SI of 0.5 and 0.25 mean, respectively, that half and 1/4 of the calcium transients are temporally overlapping. Then, the Euclidian distance between the centroid (the center-of-mass) of the ROIs was determined to correlate the SI against the distance between IHCs or between IHCs and ISCs.

Analysis of the calcium activity stack in 2D space (x, y) as a function of time (t) enabled to detect and track individual calcium waves within the Kölliker’s organ. Images were resized to 145 × 111 pixels to have a resolution of 1 pixel/μm independently of the initial binning set (2 × 2 or 4 × 4). Stacks were filtered using a 3D Gaussian smoothing kernel with 1 SD in order to remove the high-frequency noise. Calcium activity in one pixel was detected when the fluorescence ratio exceeds 1 SD of the whole activity of the Kölliker’s organ. Calcium waves were then detected as a 3D mass of pixels. For each frame, area was calculated to estimate the maximal area. Expansion rate corresponds to the ratio between the maximal area over the time from the wave detection up to its maximum area. Because the area observed may vary between the samples, the frequency of the calcium waves within the Kölliker’s organ was normalized to the observed area.

### Electrophysiology

IHCs of the apical coil were patch-clamped at their basolateral face at room temperature (22–25°C) in the perforated-patch configuration as described previously (Sendin et al., 2014). To access the plasma membrane of the IHCs, tectorial membrane, OHCs, pillar cells and IPCs were mechanically removed using glass micropipettes controlled by a micromanipulator (PatchStar, Scientifica) under an upright microscope (AxioExaminer, Zeiss). Patch pipettes were pulled from borosilicate glass capillaries (Kwik Fil, WPI) with a two-step vertical puller PIP 6 (HEKA Elektronik) and coated with silicone elastomer (Sylgard). Patch pipettes tips were first dipped during 2 min in intracellular solution devoid of amphotericin and backfilled afterwards with the intracellular solution containing (in mM): 135 KCl, 10 HEPES, 1 MgCl_2_ and 400 μg/ml amphotericin B (Calbiochem). The pH was adjusted to 7.2 with KOH and the osmolarity was between 290 and 310 mosmol/l. An EPC-10 amplifier (HEKA Elektronik) controlled by Patchmaster software (RRID:SCR_000034) was used for the action potentials measurements. All voltages were corrected for the liquid junction potential (−4.4 mV). Recordings started when the series resistance was under 30 MΩ. Spontaneous action potentials were recorded in the current clamp configuration without the injection of current (*I*_inj_ = 0 pA), low-pass filtered at 5 kHz and sampled at 25 or 40 kHz. Cells whose membrane leak current exceeded −50 pA at our standard holding potential of −74 mV were discarded.

### Experimental Design and Statistical Analysis

Cochlear turns from animals of either sex were analyzed. Mean calcium signal (amplitude, frequency, CV, duration, SI, maximal area and expansion rate) estimates present grand averages calculated from the mean estimates of individual cochlear turns. Mean estimates are expressed ± SEM and were analyzed by nonparametric Wilcoxon-Mann-Whitney two-sample rank test (two-tailed). Mean estimates of individual cochlear turns are shown in scatter plots. The analysis of the calcium activity was restricted for all the samples over the first 20 min of recordings. For the tonotopic measurement, we normalized the length of the cochlea with a value of 1 to the extreme apex and a value of 0 to the hook region. Thus, apical IHCs were located at a distance of 0.75 ± 0.02 (range, 0.69–0.83) and basal IHCs at a distance of 0.19 ± 0.02 (range, 0.11–0.28). The number of IHCs analyzed was approximately 10.7 ± 0.4 per cochlear turn. Analysis was done using Matlab (RRID:SCR_001622) and Igor Pro 7 (RRID:SCR_000325) software.

## Results

### Spontaneous Intracellular Calcium Transients Correspond to Bursts of Action Potentials in IHCs

**Figure 1:**
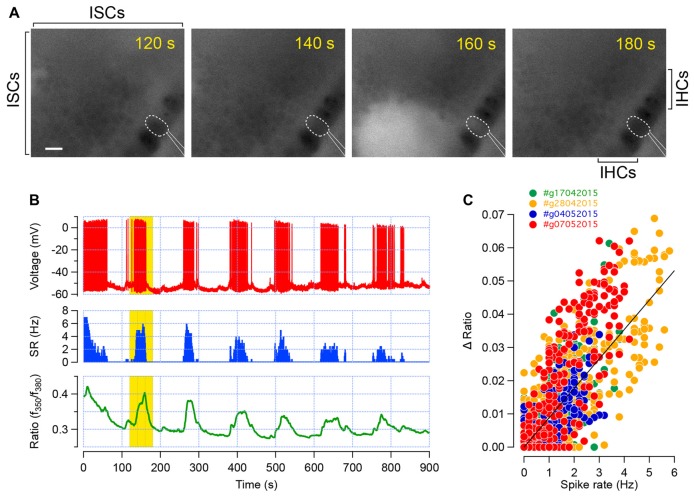
Calcium transients in inner hair cells (IHCs) correspond to trains of action potentials. **(A)** Intracellular calcium dynamic, given by the fura-2 fluorescence ratio (f350/f380), in the apical turn of a developing cochlea (P6). Each frame shows the spontaneous increase in [Ca^2+^]_i_ within the inner supporting cells (ISCs) of the Kölliker’s organ as well as in the sensory IHCs. The patch-clamped IHC is outlined in dashed white line and the patch-pipette in white lines. The increase in [Ca^2+^]_i_ within several supporting cells and in the recorded IHC is conspicuous at 160 s. Scale bar: 10 μm. **(B)** Action potential firing (red, top), spike rate (blue, middle) and fura-2 ratiometric fluorescence measurement (green, bottom) of the corresponding IHC recorded in **(A)**. Each train of action potential is temporally associated with an increase in [Ca^2+^]_i_. Yellow background indicates the corresponding time frame shown in **(A)**. **(C)** Fura-2 fluorescence ratio changes (ΔR) plot against the spike rate. Data were collected from four different recordings (three apical turns between P5 to P8 and one P7 basal turn). Each dot represents the ΔR and spiking rate average over 1 s. Linear regression fit is shown in black. Pearson’s coefficient, *R* = 0.8.

After loading the Kölliker’s organ with the calcium dye Fura2-AM, spontaneous intracellular calcium ([Ca^2+^]_i_) rises were readily observed within the ISCs and sensory IHCs (Figure 1A). To confirm whether these calcium transients in IHCs correspond to trains of action potentials, we simultaneously probed the electrical activity of the IHCs using the perforated patch-clamp technique (Figures 1A,B). In current-clamp mode (*I*_inj_ = 0 pA), the firing of the IHCs consists in trains of action potentials flanked by silent segments as previously shown (Sendin et al., 2014). In four different IHC recordings, we found out that bursts of action potentials were associated with [Ca^2+^]_i_ increase (Figure 1B). Accordingly, plotting the [Ca^2+^]_i_ rise against the spike rate demonstrated that [Ca^2+^]_i_ increases with the discharge rate (Figure 1C). Therefore, these data indicate that probing the intracellular calcium transients is a reasonable read-out of the electrical activity in developing IHCs.

### Calcium Transient Patterns in IHCs Are Mostly Invariant Along the Tonotopic Axis

**Figure 2:**
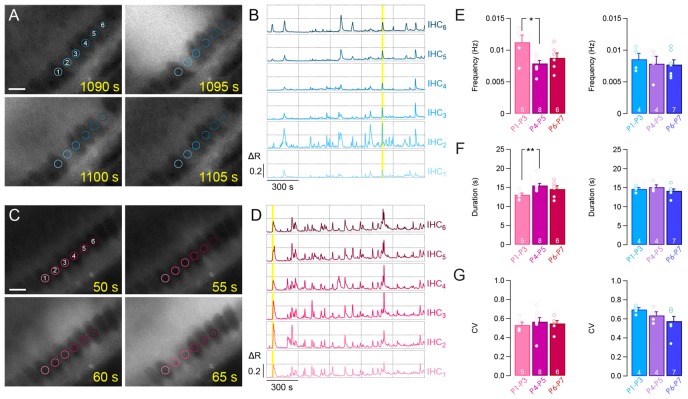
Calcium transients in IHCs over the first postnatal week. Representative examples of fura-2 f350/f380 ratio in apical **(A,B)** and basal **(C,D)** IHCs. **(A,C)** Time lapse recordings showing spontaneous [Ca^2+^]_i_ rise in several IHCs from apical **(A)** and basal **(C)** cochlear turn. For **(A)** and **(C)**, scale bar: 10 μm. **(B,D)** Calcium transients in six adjacent IHCs from **(A)** and **(C)** over 25 min of recording. Yellow background indicates the corresponding time frame shown in **(A)** and **(C)**. Regions of interest (ROIs) are indicated by colored circles onto hair cells. **(E–G)** Frequency **(E)**, duration **(F)** and coefficient of variation (CV, **G**) of the calcium transients in basal and apical IHCs during the first postnatal week. **p < 0.01 and *p < 0.05. The number of cochleas is indicated in white.

Next, we compared the spiking activity of the apical and basal IHCs over the first postnatal week (Figures 2A–D). Both apical and basal IHCs were characterized by spontaneous [Ca^2+^]_i_ rises of 10–20 s interspaced with silent periods (calcium transients duration of 14.6 ± 0.4 s and 14.1 ± 0.5 s for apical P1–P3 and P6–P7 IHCs, respectively; duration of 13.1 ± 0.5 s and 14.6 ± 0.9 s for basal P1–P3 and P6–P7 IHCs, respectively; Figure 2F). A slow increase in [Ca^2+^]_i_ baseline was sometimes observed, reflecting most probably a reduction in the calcium extrusion capacity of the sensory cells during the time-course of the experiments (Weiler et al., 2014). Frequency and duration range of the [Ca^2+^]_i_ spikes were similar over the first postnatal-week (frequency, 0.008 ± 9.10^−4^ Hz and 0.007 ± 7.10^−4^ Hz for apical P1–P3 and P6–P7 IHCs, respectively; frequency, 0.011 ± 11.10^−4^ Hz and 0.009 ± 7.10^−4^ Hz for basal P1–P3 and P6–P7 IHCs, respectively; Figure 2E). The coefficient of variation, calculated from the inter-event interval, was also in the same range over the first post-natal week and below 1, indicating the tendency of the calcium transients to occur in a regular fashion (CV, 0.69 ± 0.02 and 0.57 ± 0.05 for apical P1–P3 and P6–P7 IHCs, respectively; CV, 0.53 ± 0.03 and 0.54 ± 0.03 for basal P1–P3 and P6–P7 IHCs, respectively; Figure 2G).

**Figure 3:**
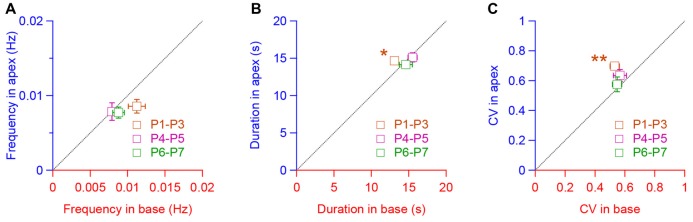
Similar pattern of activity in IHCs along the tonotopic axis.** (A–C)** Frequency **(A)**, duration **(B)** and coefficient of variation (CV, **C**) of the calcium transients in apical IHCs plot, respectively, against the frequency, duration and CV in the basal IHCs for each corresponding age (P1–P3, P4–P5, P6–P7). **p < 0.01 and *p < 0.05. Number of cochleas examined: P1–P3 base: 5, P1–P3 apex: 4, P4–P5 base: 8, P4–P5 apex: 4, P6–P7 base: 6, P6–P7 apex: 7.

We then compared the spiking pattern between apical and basal IHCs. To do so, we plotted the frequency, duration and CV means found in the apical IHCs against those in the basal hair cells for the corresponding range of age (Figures 3A–C). We found-out that the frequency, duration and CV were quite similar between the apical and basal turns except for the apical IHCs at P1–P3, which showed a larger duration (*p* = 0.046, Figure 3B) and CV (*p* = 0.008, Figure 3C). Taken together, these results suggest a comparable pattern of IHC activity along the tonotopic axis of the developing cochlea.

### Synchronous Activity Between Neighboring Cells

**Figure 4:**
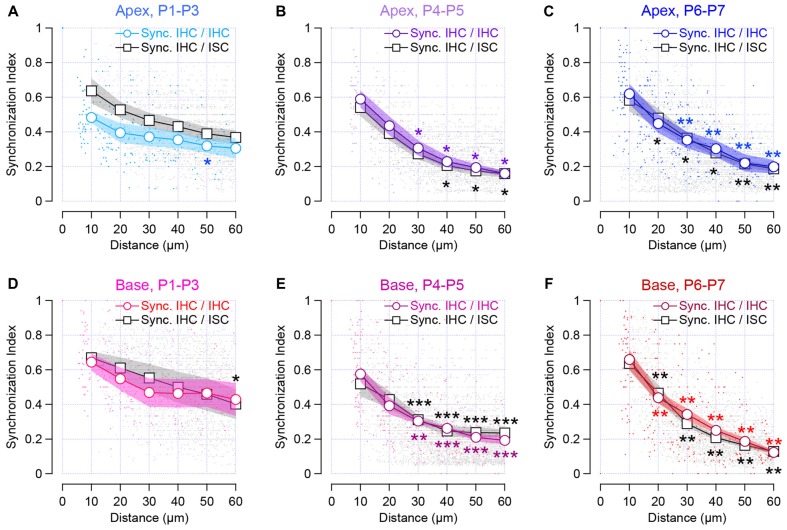
Synchronous [Ca^2+^]_i_ activity between neighboring sensory cells and non-sensory cells in the developing cochlea. The synchronous index is plot against the location of the cells. Colored symbols indicate the synchronized activity between neighboring IHCs in the apical **(A–C)** and basal cochlear turn **(D–F)**. Black and gray symbols indicate synchronous activity between sensory cells and ISCs within the Kölliker’s organ. Lines with white circles show synchronized activity binned over 10 μm distance and dots show individual data set. Stars indicate the significant difference in comparison to SI at 10 μm distance. ***p < 0.001, **p < 0.01 and *p < 0.05. Number of cochleas examined: P1–P3 base: 5, P1–P3 apex: 4, P4–P5 base: 8, P4–P5 apex: 4, P6–P7 base: 6, P6–P7 apex: 7.

Synchronized [Ca^2+^]_i_ activity was observed between two adjacent IHCs throughout the first postnatal week (SI at 10 μm distance, SI_10_, 0.48 ± 0.03 and 0.62 ± 0.06 for apical P1–P3 and P6–P7 cochleas, respectively; SI_10,_ 0.64 ± 0.05 and 0.66 ± 0.06 for basal P1–P3 and P6–P7 cochleas, respectively; Figure 4). Thus, IHCs, which are close to each other, can have a close temporal activity. Interestingly, we observed that pairs of IHC lying over a long distance still maintained a synchronized [Ca^2+^]_i_ activity at early stage of development (P1–P3) in contrast to the end of the first postnatal week (P6–P7). While most of the SI did not significantly differ from 10–60 μm distance in P1–P3 (SI_10_, 0.48 ± 0.03 vs. SI_60_, 0.3 ± 0.06, *p* = 0.057 and SI_10,_ 0.64 ± 0.05 vs. SI_60,_ 0.43 ± 0.09, *p* = 0.095 for apical and basal turns, respectively), IHCs that are distant to each other, show a significant reduction in their SI at later developmental stages (SI_10_, 0.62 ± 0.06 vs. SI_60_, 0.2 ± 0.04, *p* = 0.0025, and SI_10,_ 0.64 ± 0.05 vs. SI_60_, 0.12 ± 0.01, *p* = 0.0021 for P6–P7 apical and basal turns, respectively, Figures 4A–F).

Consistent with previous studies (Tritsch et al., 2007; Tritsch and Bergles, 2010; Wang et al., 2015), the ISCs showed a robust activity, corresponding to the propagation of spontaneous calcium transients (Figures 2A,C). When examining the temporal pattern activity between the IHC and the ISC, we found-out that IHCs and the closest neighboring supporting cells, presumably the inner border cells, tended to be synchronized (SI_10_, 0.64 ± 0.07 and 0.58 ± 0.07 for apical P1–P3 and P6–P7 cochleas, respectively; SI_10_ 0.67 ± 0.04 and 0.64 ± 0.04 for basal P1–P3 and P6–P7 cochleas, respectively, Figure 4). Although a smaller degree of synchronized activity was observed between distant IHCs and ISCs right after birth (P1–P3), it did not reach a significant difference except for 60 μm distance at the basal turn (*p* = 0.016). In contrast, ISCs located in remote areas from the hair cells show a smaller synchronization of [Ca^2+^]_i_ rise with that of IHCs at the end of the first postnatal week (SI_10_, 0.58 ± 0.07 vs. SI_60_, 0.18 ± 0.02, *p* = 0.0023 for apical P6–P7 cochleas, respectively, SI_10_, 0.64 ± 0.04, and SI_60_, 0.13 ± 0.01 *p* = 0.0021 for basal P6–P7 cochleas, respectively, Figures 4A–F). Altogether, these data suggest that neighboring IHCs together with the closest ISCs display a synchronized [Ca^2+^]_i_ activity, which tend to rapidly decrease with the distance at the end of the first postnatal week.

**Figure 5:**
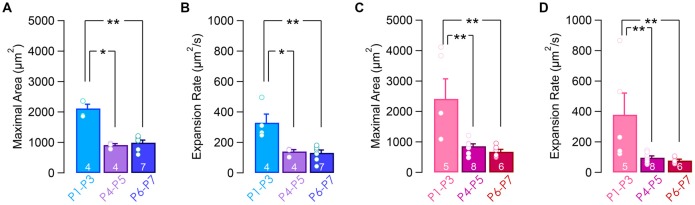
Calcium waves activity in the supporting cells within the Kölliker’s organ. Maximal area **(A,C)** and expansion rate **(B,D)** from apical **(A,B)** and basal **(C,D)** turns over the first postnatal week. **p < 0.01 and *p < 0.05. The number of cochleas is indicated in white.

The reduction in the synchronous index between remote cells over the first week of development may stem from a change in the calcium waves that propagate across the supporting cells. Indeed, the size and the velocity of the calcium waves were reduced toward the end of the first postnatal week (maximal surface area: 2118.4 ± 140.3 μm^2^ at P1–P3 vs. 993.1 ± 84 μm^2^ at P6–P7 in the apical turn, *p* = 0.004, Figure 5A, and 2415.7 ± 656.8 μm^2^ at P1–P3 vs. 680.4 ± 75.6 μm^2^ at P6–P7 in the basal turn, *p* = 0.003, Figure 5C; expansion rate: 329.7 ± 57 μm^2^.s^−1^ at P1–P3 vs. 131.7 ± 18.7 μm^2^.s^−1^ at P6–P7 in the apical turn, *p* = 0.004, Figure 5B, and 378.5 ± 142.6 μm^2^.s^−1^ at P1–P3 vs. 77.6 ± 8.9 μm^2^.s^−1^ at P6–P7 in the basal turn, *p* = 0.003, Figure 5D). Thus, the larger synchronized index between the IHCs and ISCs over long distance right after birth can be explained by larger and faster propagating calcium waves at this age.

**Figure 6:**
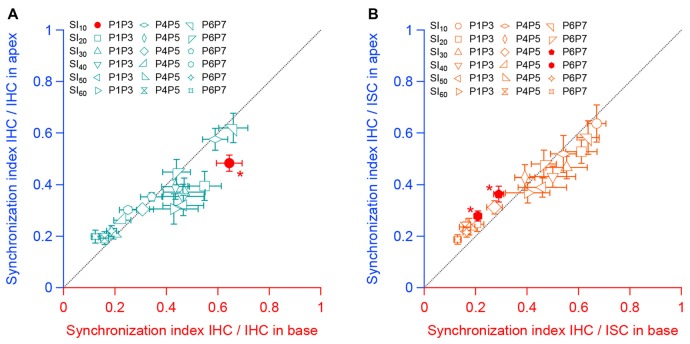
Synchronous activity between neighboring sensory cells and non-sensory cells along the tonotopic axis. **(A)** Synchronous index between apical IHCs as a function of the synchronous index between basal IHCs for each corresponding stage of development. **(B)** Synchronous index between IHCs and ISCs from the apical turn as a function of the synchronous index between IHCs and ISCs from the basal turn for each corresponding age. Black dashed line indicate the y = x regression. *p < 0.05. Number of cochleas examined: P1–P3 base: 5, P1–P3 apex: 4, P4–P5 base: 8, P4–P5 apex: 4, P6–P7 base: 6, P6–P7 apex: 7.

Finally, we compared the SI between apical and basal IHCs. To do so, we plotted the SI between the sensory cells and between IHCs and ISCs found in the apical turn against those in the basal turn for the same range of age (Figures 6A,B). We found-out that most of the SI measured at the apex did not significantly differ from those obtained in the basal turn. Taken together, these results suggest a similar mode of coordination between pairs of sensory and non-sensory cells along the base to apex gradient of the developing cochlea.

### The Inner Phalangeal Cells Influence the IHCs Synchronized Activity

The radial propagation of the calcium transients makes the ISCs and notably the inner border cells, which are surrounding the IHCs at the modiolar side, essential for the excitatory input onto the hair cells (Tritsch et al., 2007; Anselmi et al., 2008; Rodriguez et al., 2012; Dayaratne et al., 2015). However, little is known about the role of the IPCs, corresponding to the supporting cells which surround the IHCs at the pillar side. We probe the consequence of the IPCs mechanical ablation on the calcium transients in IHCs and within the ISCs. The IPCs were removed using a glass micropipette akin to the patch-clamp recordings of the hair cells, in which the IPCs have to be destroyed to access the plasma membrane.

**Figure 7:**
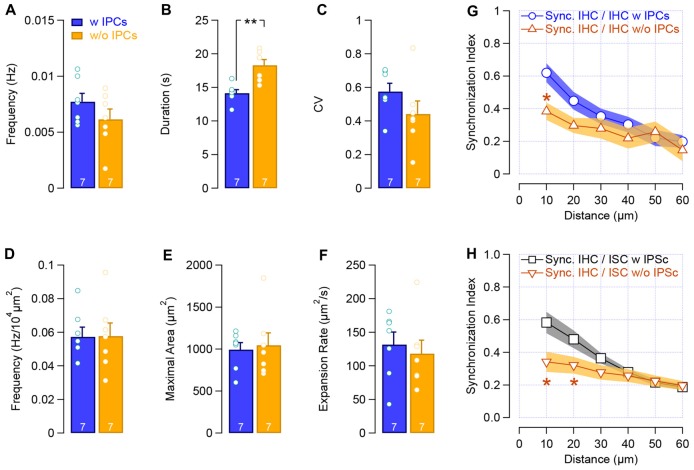
Loss of IPCs reduces the synchronized activity between neighboring cells.** (A–C)** Frequency **(A)**, duration **(B)** and CV **(C)** of the calcium transients in IHCs with IPCs (blue) and after the mechanical removal of IPCs (yellow). **(D–F)** Frequency **(D)**, maximal area **(E)** and velocity **(F)** of the calcium waves within the supporting cells of the Kölliker’s organ. **(G–H)** Synchronous activity between neighboring sensory IHCs **(G)** and between IHCs and ISCs **(H)**. **p < 0.01, *p < 0.01. The number of the cochleas (P6–P7, apical turn) is indicated in white.

The temporal distribution of the IHCs calcium transients was not notably changed following the IPCs loss (frequency: 0.007 ± 7.10^−4^ Hz vs. 0.006 ± 9.10^−4^ Hz with and w/o IPCs, respectively; CV: 0.57 ± 0.05 vs. 0.44 ± 0.08 with and w/o IPCs, respectively, Figures 7A,C), except for a slight but significant increase in the calcium transients length (duration: 14.1 ± 0.5 s vs. 18.3 ± 0.8 s with and w/o IPCs, respectively, *p* = 0.002; Figure 7B). Consistent with the initiation site of the calcium waves within the ISCs of the Kölliker’s organ, lack of IPCs did not provoke any significant difference in the propagation of the calcium waves (frequency: 0.057 ± 0.005 Hz/10^4^ μm^2^ vs. 0.057 ± 0.007 Hz/10^4^ μm^2^ with and w/o IPCs, respectively; maximal surface area: 993.1 ± 84 μm^2^ vs. 1045.5 ± 148 μm^2^ with and w/o IPCs, respectively; expansion rate: 131.7 ± 18.7 μm^2^.s^−1^ vs. 118.3 ± 20 μm^2^.s^−1^ with and w/o IPCs, respectively; Figures 7D–F). However, a reduced synchronized [Ca^2+^]_i_ activity between neighboring IHCs was observed following the mechanical ablation of the IPCs (SI_10_, 0.62 ± 0.06 vs. 0.38 ± 0.06 with and w/o IPCs, respectively, *p* = 0.011; Figure 7G) as well as between IHCs and the closest ISCs (SI_10_, 0.58 ± 0.07 vs. 0.34 ± 0.06 with and w/o IPCs, *p* = 0.017, Figure 7H). These results suggest that the IPCs do not initiate the IHCs activity but help to synchronize the adjacent sensory hair cells.

## Discussion

In this study, we showed that calcium transients, corresponding to bursts of calcium action potentials, populate the developing IHCs activity. The intracellular calcium dynamic in IHC is mostly invariant along the base to apex gradient and neighboring IHCs show a synchronized [Ca^2+^]_i_ activity.

### Calcium Transients in IHCs as a Proxy of Action Potential Bursts

During the two first postnatal weeks, IHCs fire trains of calcium action potentials with silent segments (Sendin et al., 2014; Nouvian et al., 2015). In this study, IHCs exhibit spontaneous calcium peaks, interspaced with blank periods, consistent with the firing of action potential bursts. Accordingly, the frequency and temporal distribution of these calcium spikes are, to some extent, in a similar range of those recorded in perforated patch-clamp (Sendin et al., 2014). However, the duration of the calcium transients (around 15 s) was shorter than the length of action potentials bursts measured in patch-clamp (which approximates around 50 s at the end of the first postnatal week; Sendin et al., 2014). Developing IHCs are innervated by cholinergic efferent fibers, which release acetylcholine to open the IHC calcium permeable α9α10 nicotinic receptor (Glowatzki and Fuchs, 2000; Katz et al., 2004; Roux et al., 2011). Thus, the calcium transients reflecting the calcium action potentials could have been confounded by the calcium influx through the α9α10 nicotinic receptor. This hypothesis is, however, unlikely as the calcium transients in the IHCs nicely correlate with the discharge rate of action potential. To achieve tight-seal patch-clamp recordings, the IPCs, which are the supporting cells facing the pillar side, are mechanically removed. In contrast, IPCs are left intact in our calcium imaging experiments. Therefore, the IPCs may influence the duration of the calcium transients in IHCs. Because the action potential firing in IHCs has been shown to be driven by KCl secretion from the supporting cells (Wang et al., 2015), the IPCs may be involved in the reuptake of KCl, akin the Deiters cells, which act as siphons to recycle the efflux of potassium from the OHCs (Boettger et al., 2002). Accordingly, the supporting cells of the IHCs express KCC4, required to remove potassium from the extracellular spaces (Boettger et al., 2002). Thus, the absence of IPCs may lead to a longer exposure of the IHCs to KCl. On the other hand, removing the surrounding cells would increase the KCl diffusion away from the sensory cells. In our experiments, the disruption of the IPCs tends to slightly increase the duration of the calcium transients in IHCs. However, the [Ca^2+^]_i_ rise length still remains far below the action potential bursts duration. Thus, patch-clamp recordings may affect the behavior of the IHCs. During the perforation period, in which the IHCs are held at hyperpolarized potential (−74 mV) before switching to current-clamp, the calcium channels could recover from inactivation and then be capable to sustain longer bursts of action potentials (Sendin et al., 2014).

### Invariant Pattern of Activity

Spontaneous activity in the sensory organs has been proposed to consolidate the synapses along the ascending sensory pathway (Meister et al., 1991; Wong and Oakley, 1996). In the auditory modality, the pattern of the auditory nerve fibers firing helps to refine the tonotopic map of the higher centers (Clause et al., 2014). Because the IHCs drive the spontaneous activity in the auditory nerve (Wong et al., 2013), the sensory hair cell activity should therefore be a key determinant in this maturation process. Two major mechanisms could account for the tonotopic axis specification in the auditory nuclei. In a first scenario, IHCs from basal and apical location have different pattern of activity, sustained and bursting-like spiking, respectively (Johnson et al., 2011). In this framework, auditory centers receiving a continuous input will be differentiated for high-frequencies sound encoding while others receiving patterned inputs will be specialized for low-frequencies sound encoding. In an alternative scenario, the IHC activity is rather similar along the base to apex cochlear gradient and tonotopic axis would be instructed by the coincident activation of adjacent IHCs (Tritsch and Bergles, 2010; Sendin et al., 2014). In our study, IHCs show a comparable behavior across the base to apex cochlear axis together with a large synchronous activity between neighboring IHCs. This result is therefore consistent with previous studies showing that neighboring IHCs show a similar pattern of inward currents, which have been attributed to purinergic receptor activation (Tritsch et al., 2007; Tritsch and Bergles, 2010). However, we observed that the SI between the IHCs decreases more rapidly with the distance than it has been reported earlier (Tritsch and Bergles, 2010). This difference could be explained by the threshold criteria presently used in our study, excluding weak calcium transients that may correspond to small currents in amplitude.

In our study, experiments were carried-out at room temperature to prevent any physiological-temperature degradation of the cochlear explant that may preclude the recordings of the IHC’s activity over a long period of time. Although the room-temperature condition may change the frequency and duration of the calcium transients (Sendin et al., 2014), it is unlikely that it would completely reverse the genuine behavior of the IHCs, i.e., from sustained to bursting activity. Indeed, patch-clamp recordings at room and physiological temperature indicated a similar bursting activity of the hair cells irrespective of their location along the tonotopic axis (Sendin et al., 2014).

### Synchronized Activity in the Kölliker’s Organ

ATP has been proposed to be a major key factor to release calcium from the internal stores in the neighboring supporting cells in order to regulate the IHCs excitability (Tritsch et al., 2007; Anselmi et al., 2008; Rodriguez et al., 2012; Wang et al., 2015). In addition, the activity in immature IHCs has been proposed to arise from an efflux of KCl from the neighboring supporting cells into the extracellular spaces, which surround the sensory hair cells (Wang et al., 2015). As a result, the ISCs activation triggers the depolarization of the closest IHCs to fire action potentials (Tritsch et al., 2007; Wang et al., 2015). Consistently, the IHCs and the closest ISCs facing the modiolar side (the inner border cells) show a high degree of synchronized activity. Interestingly, the coordinated activity between distant sensory and non-sensory cells varies during the first postnatal week, most likely because of the decrease in the area and velocity of the propagating calcium waves. This finding is somehow different from a previous study showing that the calcium waves area increases up to 6 days after birth (Tritsch and Bergles, 2010). Although this difference may arise from the two different species used (mouse vs. rat), further experiments are required to resolve this discrepancy.

In our experiments, the lack of IPCs does not alter the frequency of the IHCs calcium transients, excluding a crucial function of the IPCs in initiating the hair cells spiking activity. By contrast, the synchronized activity between IHCs was quite sensitive to the mechanical loss of the IPCs. It has been demonstrated that the calcium propagation within the supporting cells of the Kölliker’s organ relies on the diffusion of second messengers, such as IP3, through the connexins network expressed by the ISCs (Beltramello et al., 2005; Anselmi et al., 2008; Rodriguez et al., 2012). By diffusing the second messengers and releasing KCl in the vicinity of the hair cells, the IPCs may help to synchronize adjacent IHCs. In addition, the coordinated activity between IHCs and neighboring ISCs is reduced after the loss of the IPCs. Because of the coupling between the IPCs and the inner border cells through gap-junctions (Jagger and Forge, 2006), the mechanical destruction of the IPCs may alter the inner border cells and result in a reduced activation of the sensory cells. In this hypothesis, the frequency of the calcium transients in the IHCs should be affected, in contrast to our result. Thus, the loss of the overlapping activity between the IHCs and the closest ISCs suggests that the IPCs organize the temporal distribution of the sensory cells’ activity, most likely thought the transfer of the calcium signaling.

## Author Contributions

A-GH and J-CC equally contributed to the article. A-GH, GS, JB, J-LP and RN designed the research. A-GH, GS and RN performed the research. J-CC, JB, J-LP and RN contributed to unpublished reagents and analytic tools. A-GH, J-CC and RN analyzed data. A-GH, J-CC, GS, JB, J-LP and RN wrote the article.

## Conflict of Interest Statement

The authors declare that the research was conducted in the absence of any commercial or financial relationships that could be construed as a potential conflict of interest.
